# Supplementary material for: Round complexity in the local transformations of quantum and classical states
Source: Nat Commun. 2017 Dec 12;8:2086. doi: 10.1038/s41467-017-01887-5 (PMC5727054; doi:10.1038/s41467-017-01887-5)
Supplement: Supplementary file 1 — Supplementary Information [file 41467_2017_1887_MOESM1_ESM.pdf]

Supplementary Note 1: Gács-Körner Common Information

In what follows, we let $H(X)$ denote the Shannon entropy of a random variable X and $I(X:Y)$ the mutual information between two variables X and Y. All other notation is the same as the main text.

For a general pair of random variables XY with distribution p^{XY}, there exists a maximal common variable J_{XY} in the sense that J_{XY} can be computed exactly from either X or Y, and any other such common function of X and Y is itself a function of J_{XY}. Hence, up to relabeling, the variable J_{XY} is unique for each pair of variables XY, and Gács and Körner identify $H(J_{XY})$ as the common information of XY [1]. For values $x, x' \in \mathcal{X}$, it is not difficult to show that $J_{XY}(x) = J_{XY}(x')$ iff there exists a sequence of values

$$xy_1x_1y_2x_2\cdots x_nx' \tag{1}$$

with $x, x_1, \cdots, x_n, x' \in \mathcal{X}$ and $y_1, \cdots y_n \in \mathcal{Y}$ such that $p_{xy_1}p_{y_1x_1}p_{y_1x_2}\cdots p_{y_nx'} > 0$ [1, 2].

One can go further and introduce the maximal conditional common function [2, 3]. For three random variables XYZ, a maximal conditional common function $J_{XY|Z}$ is the collection of variables $\{J_{XY|Z=z} : z \in \mathcal{Z}\}$ with $J_{XY|Z=z}$ being a maximal common function of the conditional distribution $p^{XY|Z=z}$. The variable $J_{XY|Z}$ is again unique for every distribution p^{XYZ} up to relabeling. For all distributions considered in this paper, including the origami distributions, we will assume that some canonical ordering has be fixed (and known to all parties) so that we may speak unambiguously of the maximal common function J_{XY} and the maximal conditional common function $J_{XY|Z}$.

Let us now analyze the origami distributions in terms of the Gács-Körner common information. We first focus on random variables XYZ whose distribution is given by $\mathbf{b}^{(1,\lambda)}$. From Eq. (1) and the graphical representation of Eq. (9), we can see that x and x' satisfy $J_{XZ}(x) = J_{XZ}(x')$ iff a path connects the columns corresponding to x and x' such that movement (possibly diagonal) from one column occurs only through a common value of z. A similar rule stipulates that y and y' satisfies $J_{YZ}(y) = J_{YZ}(y')$ iff the rows corresponding to y and y' are connected by a path that only switches rows if a common value of z belongs to both rows. In Eq. (9), we see that J_{XZ} is constant (trivial) while J_{YZ} is a binary outcome with its value determined by whether $y \in \{0,1\}$ or $y \in \{2,3\}$. In a similar way, $J_{XY}(x) = J_{XY}(x')$ iff the columns of x and x' can be connected by a path that changes columns iff those columns have possible events occurring in the same row. Hence, J_{XY} is trivial for the distribution depicted in Eq. (9).

The origami distributions are constructed precisely to satisfy the following proposition, which can be proven by inspection and using simple inductive arguments following the discussion of the previous paragraph.

Proposition 1. *For any fixed value of λ, let $X^{(n)}Y^{(n)}Z^{(n)}$ denote random variables whose distribution is given by $\mathbf{b}^{(n,\lambda)}$. Then*

1. *The variable $J_{X^{(n)}Y^{(n)}}$ is trivial for all n;*
2. *For odd (resp. even) n, the variable $J_{X^{(n)}Z^{(n)}}$ (resp. $J_{Y^{(n)}Z^{(n)}}$) is trivial while $J_{Y^{(n)}Z^{(n)}}$ (resp. $J_{X^{(n)}Z^{(n)}}$) is binary;*
3. *For odd (resp. even) n, the distribution $\mathbf{b}^{(n,\lambda)}$ is equivalent (up to relabeling) to $\mathbf{b}^{(n-1,\lambda)}$ when conditioned on the value of $J_{Y^{(n)}Z^{(n)}}$ (resp. $J_{X^{(n)}Z^{(n)}}$).*
4. *$I(X^{(n)}:Y^{(n)}|Z^{(n)}) = H(J_{X^{(n)}Y^{(n)}|Z^{(n)}}|Z^{(n)}) = h(\lambda)$ for all n.*

Supplementary Note 2: The Proof of Theorem 1

Theorem 1. *For any pair (r,λ) and any $0<\lambda'\leq 1/2$, the LOPC transformation*

$$\mathbf{b}^{(r,\lambda)} \to \Phi_{\lambda'} \tag{2}$$

and the LOCC transformation

$$\rho_{\mathbf{b}}^{(r,\lambda)} \to |\Phi_{\lambda'}\rangle\langle\Phi_{\lambda'}| \tag{3}$$

are both impossible using $r-1$ rounds of communication exchanges; nor are they possible in r rounds if Alice (resp. Bob) is the first to announce when r is odd (resp. even). Conversely, for $\lambda'\leq\lambda\leq 1/2$ the transformations are possible in r rounds if Bob (resp. Alice) is the first to announce when r is odd (resp. even).

Achievability

First consider the classical case. Given Proposition 1, it is easy to see that the transformation $\mathbf{b}^{(r,\lambda)} \to \Phi_\lambda$ is possible in r rounds: each party alternates in announcing his/her common information with Eve, with Alice (resp. Bob) going first when r is even (resp. odd). With the state Φ_λ, the transformation to $\Phi_{\lambda'}$ can always be performed whenever $\lambda'<\lambda$ [4]. Such a transformation requires one-way communication, but this communication can always be included in the rth round message of the protocol. The r-round achievability in the quantum case is equivalent to the classical protocol with Alice and Bob replacing their common information announcement with the corresponding two-outcome projective measurements.

Necessity

Both transformations are clearly impossible when $\lambda'>\lambda$, which can be seen by appealing to monotonicity of LOCC/LOPC monotones. When $\lambda'>\lambda$ the so-called entanglement of formation would need to been increased in the LOCC transformation (which is not possible [5]), and the analgous conditional mutual information $I(X:Y|Z)$ would need to be increased in the LOPC transformation (which is likewise not possible [6]). Henceforth, we restrict attention to the case that $\lambda\geq\lambda'$. The proof is separated into quantum and classical parts.

The Quantum Scenario

Let us begin by introducing some new notation based on the block diagrams of $\mathbf{b}^{(r,\lambda)}$. Let $\mathcal{B}^{(r)}$ be the set of events (x,y,z) such that $\mathbf{b}_{xyz}^{(r,\lambda)}>0$. For every $k=1,\cdots,r-1$, there exists a disjoint

partitioning of $\mathcal{B}^{(r)}$ into subsets $\mathcal{B}^{(r-k)}_{j_1,\cdots,j_k}$ such that $(x,y,z) \in \mathcal{B}^{(r-k)}_{j_1,\cdots,j_k}$ if $\mathbf{b}^{(r,\lambda)}_{xyz|j_1,\cdots,j_k} > 0$, where $j_i \in \{0,1\}$ is the value of Alice's (resp. Bob's) common information with Eve given all previous values $j_1,\cdots,j_{i-1}$ when $r-(i-1)$ is even (resp. odd). In other words, the sets $\mathcal{B}^{(r-k)}_{j_1,\cdots,j_k}$ are the supports of the different sub-distributions $\mathbf{b}^{(r-k,\lambda)}$ used to build $\mathbf{b}^{(r,\lambda)}$ in the recursive construction. With a slight abuse of terminology, we will write, for instance, $x \in \mathcal{B}^{(r-k)}_{j_1,\cdots,j_k}$ if there exists some (y,z) such that $(x,y,z) \in \mathcal{B}^{(r-k)}_{j_1,\cdots,j_k}$. Note that for every z and fixed k, there exists one and only one set $\mathcal{B}^{(r-k)}_{j_1,\cdots,j_k}$ such that $z \in \mathcal{B}^{(r-k)}_{j_1,\cdots,j_k}$.

Recall that $\rho^{(r,\lambda)}_{\mathbf{b}}$ has the decomposition $\left\{\frac{1}{2^{r+1}}, |\psi^{(r,\lambda)}_z\rangle\right\}$. We will drop the superscript in the state $|\psi^{(r,\lambda)}_z\rangle$ in the proof to ease notation. The deterministic transformation $\rho^{(r,\lambda)}_{\mathbf{b}} \to |\Phi_{\lambda'}\rangle\langle\Phi_{\lambda'}|$ requires that the LOCC protocol transforms $|\psi_z\rangle \to |\Phi_{\lambda'}\rangle$ for every $|\psi_z\rangle$. Since both the initial and final states have Schmidt rank two in the transformation $|\psi_z\rangle \to |\Phi_{\lambda'}\rangle$, every local operation must either eliminate the state $|\psi_z\rangle$ or preserve its Schmidt rank. We will use this crucial fact to first argue that at the end of $r-1$ rounds on $\rho^{(r,\lambda)}_{\mathbf{b}}$ there will always be some outcome branch for which four entangled states have not been eliminated, each of them having values associated with the same block $\mathcal{B}^{(1)}_{j_1,\cdots,j_{r-1}}$. We then show that it is impossible for all of these four entangled states to be simultaneously transformed into $|\Phi_{\lambda'}\rangle$ using local operations and no communication. Therefore, there will always be some outcome branch in which an entangled pure state is not obtained.

We proceed with the following inductive argument whose validity when $k=0$ is trivial.

Inductive assumption: Along some branch at the end of round k, there exists a set $\mathcal{B}^{(r-k)}_{j_1,\cdots,j_k}$ such that $|\psi_z\rangle$ has not been eliminated for all $z \in \mathcal{B}^{(r-k)}_{j_1,\cdots,j_k}$. Let us denote the posterior states of $|\psi_z\rangle$ at this point in the protocol by $|\psi'_z\rangle$ (i.e. $|\psi'_z\rangle \propto A\otimes B|\psi_z\rangle$, where $A\otimes B$ is the full Kraus operator representing all local measurements performed up to this point of the protocol). Thus, $|\psi'_z\rangle$ is a rank-two entangled state for all $z \in \mathcal{B}^{(r-k)}_{j_1,\cdots,j_k}$. We denote $|x'\rangle \propto A|x\rangle$ and $|y'\rangle \propto B|y\rangle$ as the posterior states of $|x\rangle$ and $|y\rangle$ at this point in the protocol for all $(x,y) \in \mathcal{B}^{(r-k)}_{j_1,\cdots,j_k}$. Note that the $|x'\rangle$ and $|y'\rangle$ span the local supports of the $|\psi'_z\rangle$.

Without loss of generality, suppose that $r-k$ is even. We consider first the case when Bob is the measuring party in round $k+1$. Since $r-k$ is even, Bob and Eve do not share any common information in the distribution $\mathbf{b}^{(r-k,\lambda)}$ (see Proposition 1). Since $Srk(|\psi'_z\rangle) = Srk(|\psi_z\rangle)$ for all $z \in \mathcal{B}^{(r-k)}_{j_1,\cdots,j_k}$, an application of Proposition 2 (see below) shows that for any $\overline{y},\overline{z} \in \mathcal{B}^{(r-k)}_{j_1,\cdots,j_k}$ there exists a sequence

$$\overline{y}z_1, y_1, z_2, y_2\cdots, z_n y_n \overline{z}$$

with $y_i, z_i \in \mathcal{B}^{(r-k)}_{j_1,\cdots,j_k}$ such that

$$\langle\overline{y}'|\rho'^B_{z_1}|\overline{y}'\rangle\langle y'_1|\rho'^B_{z_1}|y'_1\rangle\langle y'_1|\rho'^B_{z_2}|y'_1\rangle\cdots\langle y'_n|\rho'^B_{\overline{z}}|y'_n\rangle > 0, \tag{4}$$

where $\rho'^B_{z_i} = \mathrm{tr}_A\,|\psi'_{z_i}\rangle\langle\psi'_{z_i}|$. Here we are using the inductive assumption that every $|\psi'_z\rangle$ is a rank-two entangled state for $z \in \mathcal{B}^{(r-k)}_{j_1,\cdots,j_k}$; since if, say, $|y_1\rangle \in supp[\rho^B_{z_1}]$, then $|y'_1\rangle \in supp[\rho'^B_{z_1}]$ when $|\psi'_z\rangle$ is entangled.

Now let $\{B_\mu\}_\mu$ be Kraus operators characterizing Bob's local measurement in round $k+1$. We argue that for every value of μ, either $\mathbb{I}\otimes B_\mu|\psi'_z\rangle = 0$ for all $z \in \mathcal{B}^{(r-k)}_{j_1,\cdots,j_k}$, or $\mathbb{I}\otimes B_\mu|\psi'_z\rangle \neq 0$ for all $z \in \mathcal{B}^{(r-k)}_{j_1,\cdots,j_k}$. Indeed, suppose there is some z for which $\mathbb{I}\otimes B_\mu|\psi'_z\rangle = 0$. This means $B_\mu|\overline{y}'\rangle = 0$ for $|\overline{y}'\rangle$ in the support of ρ'^B_z. Consider now any $\overline{z} \in \mathcal{B}^{(r-k)}_{j_1,\cdots,j_k}$. There must exist some sequence

$\overline{y}z_1, y_1, z_2, y_2 \cdots, z_n y_n \overline{z}$ such that Eq. (4) holds. The fact that $\langle \overline{y}'|\rho'^B_{z_1}|\overline{y}'\rangle > 0$ (as seen from Eq. (4)) implies that $B_\mu \rho'^B_{z_1} B_\mu^\dagger$ no longer has rank two since $B_\mu|\overline{y}'\rangle = 0$. However, every $\mathbb{I}\otimes B_\mu|\psi'_z\rangle$ either must be eliminated or have rank two; hence $B_\mu \rho'^B_{z_1} B_\mu^\dagger$ must vanish. From the second term in Eq. (4), we see that $\langle y'_1|\rho'^B_{z_1}|y'_1\rangle > 0$, which means that $B_\mu|y'_1\rangle = 0$ in order for $B_\mu \rho'^B_{z_1} B_\mu^\dagger$ to vanish. Continuing along the sequence of Eq. (4) and repeating this argument, we will eventually reach $\rho'^B_{\overline{z}}$, which also must be eliminated by B_μ. Since $\overline{z}$ was arbitrary, we have established that $\mathbb{I}\otimes B_\mu|\psi'_z\rangle = 0$ for some $z \in \mathcal{B}^{(r-k)}_{j_1,\cdots,j_k}$ implies that $\mathbb{I}\otimes B_\mu|\psi'_z\rangle = 0$ for all $z \in \mathcal{B}^{(r-k)}_{j_1,\cdots,j_k}$. Finally, since $\{B_\mu\}_\mu$ is a complete measurement, there must exist at least one outcome $\overline{\mu}$ such that $\mathbb{I}\otimes B_{\overline{\mu}}|\psi'_{\overline{z}}\rangle \neq 0$ for some $\overline{z} \in \mathcal{B}^{(r-k)}_{j_1,\cdots,j_k}$. Let $j_{k+1} \in \{0,1\}$ be such that $\overline{z} \in \mathcal{B}^{(r-k-1)}_{j_1,\cdots,j_k,j_{k+1}}$. Since $\mathcal{B}^{(r-k-1)}_{j_1,\cdots,j_k,j_{k+1}}$ is just a subset of $\mathcal{B}^{(r-k)}_{j_1,\cdots,j_k}$, we have that $\mathbb{I}\otimes B_\mu|\psi'_z\rangle \neq 0$ for all $z \in \mathcal{B}^{(r-k-1)}_{j_1,\cdots,j_k,j_{k+1}}$. This verifies the inductive assumption when Bob is measuring in round $k+1$.

We now consider the case when Alice is the measuring party in round $k+1$ with $r-k$ being even as before. In this case, Alice and Eve share one bit of common information in the distribution $\mathbf{b}^{(r-k,\lambda)}$. However, this information simply specifies whether a given $z \in \mathcal{B}^{(r-k)}_{j_1,\cdots,j_k}$ either belongs to $\mathcal{B}^{(r-k-1)}_{j_1,\cdots,j_k,0}$ or $\mathcal{B}^{(r-k-1)}_{j_1,\cdots,j_k,1}$. Most importantly, Alice has no common information with Eve for values (x,z) within either $\mathcal{B}^{(r-k-1)}_{j_1,\cdots,j_k,0}$ or $\mathcal{B}^{(r-k-1)}_{j_1,\cdots,j_k,1}$. Hence by repeating the previous argument within those sub-blocks, we have that for her measurement $\{A_\mu\}_\mu$ either $A_\mu\otimes\mathbb{I}|\psi'_z\rangle = 0$ for all $z \in \mathcal{B}^{(r-k-1)}_{j_1,\cdots,j_k,0}$, or $A_\mu\otimes\mathbb{I}|\psi'_z\rangle \neq 0$ for all $z \in \mathcal{B}^{(r-k-1)}_{j_1,\cdots,j_k,0}$; and likewise $A_\mu\otimes\mathbb{I}|\psi'_z\rangle = 0$ for all $z \in \mathcal{B}^{(r-k-1)}_{j_1,\cdots,j_k,1}$, or $A_\mu\otimes\mathbb{I}|\psi'_z\rangle \neq 0$ for all $z \in \mathcal{B}^{(r-k-1)}_{j_1,\cdots,j_k,1}$. There must be at least one outcome $\overline{\mu}$ with $A_{\overline{\mu}}\otimes\mathbb{I}|\psi'_{\overline{z}}\rangle \neq 0$ for some $\overline{z} \in \mathcal{B}^{(r-k)}_{j_1,\cdots,j_k}$. Let $j_{k+1} \in \{0,1\}$ be such that $\overline{z} \in \mathcal{B}^{(r-k-1)}_{j_1,\cdots,j_k,j_{k+1}}$. This verifies the inductive assumption for round $k+1$.

Having proven the inductive assertion, we now apply it to a hypothetical $(r-1)$-round LOCC protocol that transforming $\rho^{(r,\lambda)}_{\mathbf{b}}$ into $|\Phi_{\lambda'}\rangle$. At the end of this protocol, there must exist some set $\mathcal{B}^{(1)}_{j_1,\cdots,j_{r-1}}$ such that the posterior probability of the state $|\psi_z\rangle$ is nonzero for every $\mathcal{B}^{(1)}_{j_1,\cdots,j_{r-1}}$. But since there are no more rounds left in the protocol, each of these states must be locally convertible into the target state $|\Phi_{\lambda'}\rangle$ with no further communication. Proposition 3 below shows that this is not possible, and therefore the hypothetical $(r-1)$-round protocol performing the desired transformation does not exist.

The final part of the proof is to show that the transformation is impossible in r rounds if Alice (resp. Bob) is the first to announce when r is odd (resp. even). The reasoning follows exactly along the lines of the proceeding argument. Consider the case when Alice is announcing first and r is odd. In this case she initially shares no common information with Eve. Therefore, her measurement is unable to eliminate any of the $|\psi_z\rangle$ and so at the end of round 1, all values of z still belong to $\mathcal{B}^{(r)}$. We can then repeat the above argument except with the modified inductive assumption: Along some branch at the end of round k there exists a set $\mathcal{B}^{(r-(k-1))}_{j_1,\cdots j_{k-1}}$ such that $|\psi_z\rangle$ has not been eliminated for all $z \in \mathcal{B}^{(r-(k-1))}_{j_1,\cdots j_{k-1}}$. Proceeding for r rounds again leaves Alice and Bob with at least one block $\mathcal{B}^{(1)}_{j_1,\cdots j_{r-1}}$ with no states eliminated. From Proposition 3, the transformation cannot be completed.

We now prove the two main propositions referenced in the above proof. The first essentially says that when embedding a probability distribution into a tripartite quantum states, common information cannot be generated between Eve and any one of the other two parties, even with just a nonzero probability.

Proposition 2. *Let $\{|\psi_z\rangle^{AB}\}_{\mathcal{Z}}$ be a collection of bipartite states for Alice and Bob's systems and Z a random variable ranging over $\mathcal{Z}$ with distribution p^Z. Suppose that $H(J_{XZ}) = H(J_{YZ}) = 0$ for distributions*

$$p^{XZ}_{xz} = \langle x|(\mathrm{tr}_B\,|\psi_z\rangle\langle\psi_z|)|x\rangle \qquad \text{and} \qquad p^{YZ}_{yz} = \langle y|(\mathrm{tr}_A\,|\psi_z\rangle\langle\psi_z|)|y\rangle. \tag{5}$$

Let A and B be any pair of operators such that $Srk(|\psi'_z\rangle) = Srk(|\psi_z\rangle)$ for all z, where $Srk(\cdot)$ is the Schmidt rank of the given state and $|\psi'_z\rangle = \frac{A\otimes B|\psi_z\rangle}{\sqrt{\langle\psi_z|A^\dagger A\otimes B^\dagger B|\psi_z\rangle}}$. Then for any $\overline{x}, \overline{z}$ there exists a sequence $(x_i, z_i)_i$ such that

$$\langle\overline{x}'|\rho'^A_{z_1}|\overline{x}'\rangle\langle x'_1|\rho'^A_{z_1}|x'_1\rangle\langle x'_1|\rho'^A_{z_2}|x'_1\rangle\cdots\langle x'_n|\rho'^A_{\overline{z}}|x'_n\rangle > 0, \tag{6}$$

where $\rho'^A_i = \mathrm{tr}_B\,|\psi'_z\rangle\langle\psi'_z|$ and $|x'\rangle = \frac{|x\rangle}{\sqrt{\langle x|A^\dagger A|x\rangle}}$. An analogous statement holds for any pair of values $\overline{y}, \overline{z}$.

Proof. By the assumption that $H(J_{XZ}) = 0$, we have that for any $\overline{x}, \overline{z}$ there exists a sequence $(x_i, z_i)_i$ such that

$$p^{XZ}_{\overline{x},z_1} p^{XZ}_{x_1,z_1} p^{XZ}_{x_1,z_2}\cdots p^{XZ}_{x_n,z_n} p^{XZ}_{x_n,\overline{z}} > 0. \tag{7}$$

The essential observation is that if $Srk(A\otimes B|\psi_z\rangle) = Srk(|\psi_z\rangle)$, then

$$\langle x|(\mathrm{tr}_B\,|\psi_z\rangle\langle\psi_z|)|x\rangle > 0 \qquad \Rightarrow \qquad \langle x|A^\dagger(\mathrm{tr}_B\,A\otimes B|\psi_z\rangle\langle\psi_z|A^\dagger\otimes B^\dagger)A|x\rangle > 0. \tag{8}$$

Indeed, the first inequality says that (i) $|x\rangle \in supp\,(\mathrm{tr}_B\,|\psi_z\rangle\langle\psi_z|)$. Since $A\otimes B$ does not decrease the rank of $|\psi_z\rangle$, this means that (ii) $supp\,(\mathrm{tr}_B\,|\psi_z\rangle\langle\psi_z|) = supp\,\left(\mathrm{tr}_B\,\mathbb{I}\otimes B|\psi_z\rangle\langle\psi_z|\mathbb{I}\otimes B^\dagger\right)$, and (iii) $A|x\rangle \neq 0$. Combining facts (i)–(iii) gives that $A|x\rangle \in supp\,\left(\mathrm{tr}_B\,A\otimes B|\psi_z\rangle\langle\psi_z|A^\dagger\otimes B^\dagger\right)$. By interchanging x and y in this argument, an analogous inequality to (6) is proven for any pair $\overline{y}, \overline{z}$. □

The second proposition provides the final ingredient in the above proof. Up to relabeling, the events in $\mathcal{B}^{(1)}_{j_1,\cdots,j_{r-1}}$ corresponds to the events in the support of $\mathbf{b}^{(1,\lambda)}$. For reference, we reproduce the diagram depicting $\mathbf{b}^{(1,\lambda)}$:

$$\mathbf{b}^{(1,\lambda)} = \quad \begin{array}{cc|cccc} & & & X & & \\ & & 0 & 1 & 2 & 3 \\ \hline & 0 & 0 & \cdot & \cdot & 1 \\ & 1 & \cdot & 0 & 1 & \cdot \\ Y & 2 & 2 & 3 & \cdot & \cdot \\ & 3 & \cdot & \cdot & 3 & 2 \quad Z \end{array} \tag{9}$$

Proposition 3. *Let $\{|\psi_z\rangle\}_{z=0}^3$ be the four entangled states obtained by the embedding of $\mathbf{b}^{(1,\lambda)}$. Let $\{|\psi'_z\rangle\}_{z=0}^3$ be the resulting four states at the end of one branch in an LOCC protocol, all of them being rank-two entangled. Then it is not possible to transform each of the states into $|\Phi_{\lambda'}\rangle$ using just local operations with no further communication.*

Proof. Let $A\otimes B$ be the measurement operators corresponding to this branch in the protocol. In other words $|\psi'_z\rangle \propto A\otimes B|\psi_z\rangle$, and we can write

$$|\psi'_0\rangle \propto \left(|0'\rangle^A|0'\rangle^B + |1'\rangle^A|1'\rangle^B\right) \tag{10}$$

$$|\psi'_1\rangle \propto \left(|2'\rangle^A|0'\rangle^B + |3'\rangle^A|1'\rangle^B\right) \tag{11}$$

$$|\psi'_2\rangle \propto \left(|0'\rangle^A|2'\rangle^B + |3'\rangle^A|3'\rangle^B\right) \tag{12}$$

$$|\psi'_3\rangle \propto \left(|1'\rangle^A|2'\rangle^B + |2'\rangle^A|3'\rangle^B\right) \tag{13}$$

where $|i'\rangle^A \propto A|i\rangle^A$ and $|j'\rangle^B \propto B|j\rangle^B$ for all j. The local operation will consist in each party performing a local measurement and unitary rotation. Since no communication is allowed, the target state $|\Phi_{\lambda'}\rangle$ must be obtained after every possible combination of outcomes. Let B_0 be one of Bob's measurement operators that doesn't eliminate either $|\psi'_0\rangle^B$ or $|\psi'_1\rangle^B$, and let B_1 be one of Bob's measurement operators that doesn't eliminate either $|\psi'_2\rangle^B$ or $|\psi'_3\rangle^B$ (such operators must exist). Its clear that by applying B_0 to the first pair and B_1 to the second pair, the form of Eqns. (10)–(13) will stay the same. Likewise, there must exist some operator A_0 of Alice that does not eliminate either $|0'\rangle^A$ or $|1'\rangle^A$. Additionally, this operator cannot eliminate either $|3'\rangle^A$ or $|2'\rangle^A$, or else the ranks of $|\psi'_2\rangle$ and $|\psi'_3\rangle$ will drop to one respectively. Thus, applying operator A_0 to the states will not change their form either.

Without loss of generality then, we can assume that each of the $|\psi'_z\rangle$ are nonzero, proportional to $|\Phi_{\lambda'}\rangle$, and therefore proportional to each other. The linear independence of $|0'\rangle^B$ and $|1'\rangle^B$ in Eqns. (10) and (11) implies that $|2'\rangle^A \propto |0'\rangle^A$ and $|3'\rangle^A \propto |1'\rangle^A$. Eq. (10) and (11) then give $|2'\rangle^B \propto |0'\rangle^B$ and $|3'\rangle^B \propto |1'\rangle^B$. However, this forces Eq. (13) to have the form $\alpha|1'\rangle^A|0'\rangle^B + \beta|0'\rangle^A|1'\rangle^B$ which contradicts the fact that it is proportional to $|0'\rangle^A|0'\rangle^B + |1'\rangle^A|1'\rangle^B$.

□

The Classical Scenario

In this section we will prove that the corresponding LOPC transformation $\mathbf{b}^{(r,\lambda)} \to \Phi_{\lambda'}$ is not possible in $r-1$ rounds. For notational clarity, we let $X_nY_nZ_n$ denote random variables that are jointly distributed according to $\mathbf{b}^{(n,\lambda)}$. Our goal here is to reproduce the quantum proof except in terms of an LOPC transformation of $\mathbf{b}^{(r,\lambda)}$. In the proof above, we made heavy use of the Schmidt rank of a bipartite pure state. As presented in the main text, the analog of the Schmidt rank for classical distributions is the secrecy rank.

For the simple structure of the origami distributions, the secrecy rank of the conditional distribution $p^{X_nY_n|Z_n=z}$ is equivalent to the number of events having nonzero probability given $Z_n = z$. Since $\mathbf{b}^{(r,\lambda)} \to \Phi_{\lambda'}$ is a deterministic transformation, then for every z, the conditional distribution $p^{X_rY_r|Z_r=z}$ must be transformed into $\Phi_{\lambda'}$ with probability one. Therefore, with both $p^{X_rY_r|Z_r=z}$ and $\Phi_{\lambda'}$ having secrecy rank two, monotonicity of the secrecy rank implies that every local operation must either "eliminate" the distribution $p^{X_rY_r|Z_r=z}$ or preserve its secrecy rank. In other words, for every sequence of messages $m_{\leq k} = (m_1, \cdots, m_k)$ either $p_{z|m_{\leq k}} = 0$ or both events having nonzero events in the original distribution $p^{X_rY_r|Z_r=z}$ still have nonzero posterior probability when given messages $m_{\leq k}$. This is analogous to the Schmidt rank condition we have in the quantum case.

With this connection of rank preservation established, we can now run the exact same inductive as in the quantum proof above. Hence, we can conclude that in any LOPC protocol transforming $\mathbf{b}^{(r,\lambda)} \to \Phi_{\lambda'}$: Along some branch at the end of every round k, there exists a set $\mathcal{B}^{(r-k)}_{j_1,\cdots,j_k}$ such that $p^{X_rY_r|Z_r=z}$ has not been eliminated for all $z \in \mathcal{B}^{(r-k)}_{j_1,\cdots,j_k}$. Consequently, after $r-1$ rounds, there will be some set of events $\mathcal{B}^{(1)}_{j_1,\cdots,j_{r-1}}$ corresponding to the block structure of $\mathbf{b}^{(1,\lambda)}$, except with the events having possibly different nonzero posterior probabilities than at the start of the protocol. The crucial point, however, is that no events in this set have been eliminated at the end of the $r-1$ rounds. It is very easy to see that Alice and Bob then share no common information along this branch of the protocol, and therefore the only perfectly correlated variable that they can agree on is a trivial one. Indeed, recall that non-trivial common information exists if the events with nonzero probability form disjoint blocks in the distribution; from Eq. (9) we see this is not possible when all events have a nonzero probability. Thus, we have proven that $r-1$ rounds of LOPC

are not sufficient to complete the transformation $\rho_{\mathbf{b}}^{(r,\lambda)} \to |\Phi_{\lambda'}\rangle\langle\Phi_{\lambda'}|$. When Alice (resp. Bob) is the first to announce for r is odd (resp. even), impossibility of the transformation in r rounds can be argued just as in the quantum case above.

□

Supplementary Note 3: Robustness of Theorem 1

We now offer a strengthening of Theorem 1 in terms of relaxing to ϵ-approximate transformations. Let $D_{tr}(\rho,\sigma) = \frac{1}{2}||\rho - \sigma||_1$ denote the trace distance between density operators ρ and σ, in which $||\omega||_1 = \text{tr}\sqrt{\omega^\dagger\omega}$. The trace distance is a generalization of the classical variational distance between probability distributions, the latter being defined by $d(p^{XYZ}, p^{X'Y'Z'}) := d(\rho,\sigma)$ where $\rho = \sum_{xyz} p_{xyz}^{XYZ}|xyz\rangle\langle xyz|$ and $\sigma = \sum_{xyz} p_{xyz}^{X'Y'Z'}|xyz\rangle\langle xyz|$.

Our addition to Theorem 1 then says the following.

Proposition 4. *For any pair (r,λ) and any $0 < \lambda' \leq 1/2$, there exists some $\epsilon > 0$ such that any $(r-1)$-round LOPC transformation $\mathbf{b}^{(r,\lambda)} \to p^{\widehat{X}\widehat{Y}ZM}$ and any $(r-1)$-round LOCC transformation $\rho_{\mathbf{b}}^{(r,\lambda)} \to \rho^{AB}$ must respectively satisfy*

$$D_{tr}\left(p^{\widehat{X}\widehat{Y}ZM},\ \Phi_{\lambda'}p^{ZM}\right) > \epsilon, \tag{14}$$

$$D_{tr}\left(\rho^{AB},\ |\Phi_{\lambda'}\rangle\langle\Phi_{\lambda'}|^{AB}\right) > \epsilon. \tag{15}$$

Without loss of generality it can be assumed that $|\widehat{X}| = |\widehat{Y}| = 2$ and that ρ^{AB} is a two-qubit density matrix. The reason is that since $\Phi_{\lambda'}$ ranges over a set of size 2×2, Alice and Bob can always map variables $\widehat{X}$ and $\widehat{Y}$ into binary-outcome variables without increasing the trace norm. Analogous reasoning allows us to assume that ρ^{AB} is a two-qubit state.

Eq. (15) then follows immediately from the fact that the set of $(r-1)$-round LOCC maps transforming $\mathbf{b}^{(r,\lambda)}$ into a two-qubit density matrix is compact [7]. Therefore if $D_{tr}(\rho^{AB},\ |\Phi_{\lambda'}\rangle\langle\Phi_{\lambda'}|^{AB})$ could be made arbitrarily small, then there must exist an $(r-1)$-round protocol for which $D_{tr}(\rho^{AB},\ |\Phi_{\lambda'}\rangle\langle\Phi_{\lambda'}|^{AB}) = 0$, which would contradict Theorem 1.

We cannot immediately apply the same reasoning to Eq. (14). While we can assume that $\widehat{X}$, $\widehat{Y}$, and Z are all bounded variables in the distribution $p^{\widehat{X}\widehat{Y}ZM}$, the communication M need not be bounded and we therefore do not have compactness as in the LOCC case. However, this can easily be remedied by the following lemma which allows us to bound the communication without loss of generality for the task of secrecy transformations.

Lemma 1. *Suppose there exists an r-round LOPC protocol achieving the transformation $p^{XYZ} \to p^{\widehat{X}\widehat{Y}ZM}$ with Alice (resp. Bob) announcing in odd (resp. even) rounds. Then there exists an r-round LOPC transformation $p^{XYZ} \to p^{\widehat{X}\widehat{Y}ZM'}$ for which the public messages $M' = (M'_1, M'_2, \cdots, M'_r)$ satisfy*

$$M'_t - XM'_{<t} - YZ \quad \text{for odd } t \in \{1,\cdots,r\}, \qquad \widehat{X} - XM' - YZ, \tag{16}$$

$$M'_t - YM'_{<t} - XZ \quad \text{for even } t \in \{1,\cdots,r\}, \qquad \widehat{Y} - YM' - XZ, \tag{17}$$

$$D_{tr}\left(p^{\widehat{X}\widehat{Y}ZM'},\ p^{\widehat{X}\widehat{Y}}p^{ZM'}\right) = D_{tr}\left(p^{\widehat{X}\widehat{Y}ZM},\ p^{\widehat{X}\widehat{Y}}p^{ZM}\right), \tag{18}$$

as well as the cardinality bounds

$$|M'_1| = |X| + 2 \tag{19}$$

$$|M'_t| = |X||M'_{<t}| + 2 \quad \text{for odd } t \in \{2,\cdots,r\} \tag{20}$$

$$|M'_t| = |Y||M'_{<t}| + 2 \quad \text{for even } t \in \{2,\cdots,r\}. \tag{21}$$

Proof. Our goal is to construct a protocol in which the original message sequence M is replaced by M' so the above constraints are satisfied. We proceed by induction on the round number. Suppose that for odd t we have constructed an r-round LOPC protocol that accomplishes the transformation $p^{XYZ} \to p^{\widehat{X}\widehat{Y}ZM'_1\cdots M'_{t-1}M_t\cdots M_r}$ with the M'_i satisfying Eqns. (16)–(18) and the cardinality bounds (19)–(21). The Markov chain condition (16) allows us to write

$$p^{\widehat{X}\widehat{Y}ZM'_1\cdots M'_{t-1}M_t\cdots M_r} = p^{M_t} p^{XM'_{<t}|M_t} p^{YZ|XM'_{<t}} p^{\widehat{X}\widehat{Y}M_{>t}|XYZM_tM'_{<t}} \tag{22}$$

where $M'_{<t} = M_1, \cdots, M'_{t-1}$. We further decompose

$$p^{XM'_{<t}} = \sum_{m_t} p^{XM'_{<t}|M_t=m_t} p^{M_t}_{m_t}, \tag{23}$$

$$D_{tr}\left(p^{\widehat{X}\widehat{Y}ZM},\ p^{\widehat{X}\widehat{Y}}p^{ZM}\right) = \sum_{m_t} D_{tr}\left(p^{\widehat{X}\widehat{Y}ZM'_{<t}M_{<t}|M_t=m_t},\ p^{\widehat{X}\widehat{Y}}p^{ZM'_{<t}M_{>t}|M_t=m_t}\right) p^{M_t}_{m_t}. \tag{24}$$

These encompass $|X||M'_{<t}|+1$ total constraints on the distribution p^{M_t}. Hence by Carathéodory's Theorem, there exists a random variable M'_t with cardinality $|M'_{<t}| \leq |X||M'_{<t}|+2$ and probabilities $p^{M'_t}_{m_t}$ that satisfy Eqns. (23) and (24). Futhermore, by Eq. (22) we have

$$p^{\widehat{X}\widehat{Y}ZM'_1\cdots M'_{t-1}M'_t\cdots M_r} = p^{M'_t} p^{XM'_{<t}|M'_t} p^{YZ|XM'_{<t}} p^{\widehat{X}\widehat{Y}M_{>t}|XYZM'_tM'_{<t}}. \tag{25}$$

Hence we have verified the inductive assumption and continuing for all r rounds proves the lemma. □

To apply Lemma 1, suppose that for every $\epsilon > 0$ there exists an $(r-1)$-round LOPC transformation $\mathbf{b}^{(r,\lambda)} \to p^{\widehat{X}\widehat{Y}ZM}$ for which $D_{tr}(p^{\widehat{X}\widehat{Y}ZM},\ \Phi_{\lambda'}p^{ZM}) < \epsilon$. Then by Lemma 1 there is a bounded communication protocol $\mathbf{b}^{(r,\lambda)} \to p^{\widehat{X}\widehat{Y}ZM'}$ that satisfies

$$\begin{aligned}
D_{tr}(p^{\widehat{X}\widehat{Y}ZM'},\ \Phi_{\lambda'}p^{ZM'}) &\leq D_{tr}(p^{\widehat{X}\widehat{Y}ZM'},\ p^{\widehat{X}\widehat{Y}}p^{ZM'}) + D_{tr}(p^{\widehat{X}\widehat{Y}}p^{ZM'},\ \Phi_{\lambda'}p^{ZM'}) \\
&\leq D_{tr}(p^{\widehat{X}\widehat{Y}ZM},\ p^{\widehat{X}\widehat{Y}}p^{ZM}) + \epsilon \\
&\leq D_{tr}(p^{\widehat{X}\widehat{Y}ZM},\ \Phi_{\lambda'}p^{ZM}) + D_{tr}(\Phi_{\lambda'}p^{ZM},\ p^{\widehat{X}\widehat{Y}}p^{ZM}) + \epsilon \\
&\leq 3\epsilon,
\end{aligned} \tag{26}$$

where we have repeatedly used the assumption $D_{tr}(p^{\widehat{X}\widehat{Y}ZM},\ \Phi_{\lambda'}p^{ZM}) < \epsilon$ along with Eq. (18). However, compactness now holds on the set of $(r-1)$-round protocols satisfying the communication bounds of Eqns. (19)–(21). Hence, there must exist an $(r-1)$-round protocol for which $D_{tr}(p^{\widehat{X}\widehat{Y}ZM},\ \Phi_{\lambda'}p^{ZM}) = 0$, an impossibility by Theorem 1. This completes the proof of Proposition 4.

Lemma 2 (Carathéodory's Theorem, cf. [8]). *Let S be a subset of $\mathbb{R}^n$ and $conv(S)$ its convex hull. Then any $x \in conv(S)$ can be expressed as a convex combination of at most $n+1$ elements of S.*

As it is applied in the proof of Lemma 1, S consists of $(|\mathcal{M}'_{<t}||\mathcal{X}|+2)$-component vectors with the components of each vector given by Eqns. (23) and (24) for some distribution $p^{M_t}_{m_t}$.

Supplementary Note 4: Monotonicity of the Secrecy Rank

Theorem 2 (restated). The secrecy rank is a stochastic LOPC (SLOPC) monotone.

Proof. If one of the parties, say Alice, locally generates a message M, then the resulting distribution is p^{XYZM} with secrecy rank given by

$$Srk[p^{XY(ZM)}] = \min_{XM-ZMW-YM} \max_{z,m} |p^{W|Z=z,M=m}|. \tag{27}$$

For distribution p^{XYZ}, let W_0 be any variables such that $Srk[p^{XYZ}] = \max_z |p^{W_0|Z=z}|$, and suppose that Alice generates a public message M. This implies that $MX - W_0Z - Y \Rightarrow X - MW_0Z - Y \Rightarrow MX - MW_0Z - MY$. Hence, the secrecy rank of the distribution after Alice's message satisfies

$$\begin{aligned} Srk[p^{XY(ZM)}] &= \min_{XM-ZMW-YM} \max_{z,m} |p^{W|Z=z,M=m}| \\ &= \min_{X-ZMW-YM} \max_{z,m} |p^{W|Z=z,M=m}| \\ &\leq \max_{z,m} |p^{W_0|Z=z,M=m}| \\ &\leq \max_{z} |p^{W_0|Z=z}| = Srk[p^{XYZ}]. \end{aligned} \tag{28}$$

This chain of inequalities shows that the Schmidt rank is an LOPC monotone. However, an even stronger statement can be made since $\max_{z,m} |p^{W|Z=z,M=m}| \geq \max_z |p^{W|Z=z,M=m'}|$ for any fixed message m'. Hence from Eq. (28) we can conclude that

$$Srk[p^{XYZ}] \geq Srk[p^{XY(ZM)}|M = m'] \quad \forall m' \in \mathcal{M}. \tag{29}$$

This shows that the secrecy rank cannot be increased even when conditioned on just a single message; in other words, with zero probability can the secrecy rank be increased by LOPC.

□

Supplementary References

[1] P. Gács and J. Körner. Common information is far less than mutual information. *Problems of Control and Information Theory*, 2(2):149, 1973.

[2] Eric Chitambar, Benjamin Fortescue, and Min-Hsiu Hsieh. Distributions attaining secret key at a rate of the conditional mutual information. In *Advances in Cryptology – CRYPTO 2015*, volume 9216, pages 443–462. Springer Berlin Heidelberg, 2015.

[3] Eric Chitambar, Ben Fortescue, and Min-Hsiu Hsieh. Classical analog to entanglement reversibility. *Phys. Rev. Lett.*, 115:090501, Aug 2015.

[4] Daniel Collins and Sandu Popescu. Classical analog of entanglement. *Phys. Rev. A*, 65:032321, 2002.

[5] Charles H. Bennett, David P. DiVincenzo, John A. Smolin, and William K. Wootters. Mixed-state entanglement and quantum error correction. *Phys. Rev. A*, 54(5):3824–3851, Nov 1996.

[6] Imre Csiszár and Janos Körner. *Information Theory: Coding Theorems for Discrete Memoryless Systems*. Cambridge University Press, Cambridge, UK, 2011.

[7] Eric Chitambar, Debbie Leung, Laura Maninska, Maris Ozols, and Andreas Winter. Everything you always wanted to know about locc (but were afraid to ask). *Communications in Mathematical Physics*, 328(1):303–326, 2014.

[8] R. Tyrell Rockafellar. *Convex analysis*. Princeton Mathematical Series. Princeton University Press, 1996.
